# Texture Analysis of 68Ga-DOTATOC PET/CT Images for the Prediction of Outcome in Patients with Neuroendocrine Tumors

**DOI:** 10.3390/biomedicines13061286

**Published:** 2025-05-23

**Authors:** Sara Pellegrino, Mariarosaria Panico, Roberto Bologna, Rocco Morra, Alberto Servetto, Roberto Bianco, Silvana Del Vecchio, Rosa Fonti

**Affiliations:** 1 Department of Advanced Biomedical Sciences, University Federico II, 80131 Naples, Italy; sara.pellegrino@unina.it (S.P.); robertobologna@hotmail.it (R.B.); delvecc@unina.it (S.D.V.); 2 Institute of Biostructures and Bioimages, National Research Council, 80145 Naples, Italy; mariarosaria.panico@cnr.it; 3 Department of Clinical Medicine and Surgery, University Federico II, 80131 Naples, Italy; rocco.mor4@gmail.com (R.M.); alberto.servetto@unina.it (A.S.); roberto.bianco@unina.it (R.B.)

**Keywords:** texture analysis, 68Ga-peptide PET/CT, neuroendocrine tumors, prognosis

## Abstract

**Objectives**: The aim of our study is to evaluate whether texture analysis of 68Ga-DOTATOC PET/CT images can predict clinical outcome in patients with neuroendocrine tumors (NET). **Methods**: Forty-seven NET patients who had undergone 68Ga-DOTATOC PET/CT were studied. Primary tumors were localized in the gastroenteropancreatic (n = 35), bronchopulmonary (n = 8), and other (n = 4) districts. NET lesions were segmented using an automated contouring program and subjected to texture analysis, thus obtaining the conventional parameters SUVmax and SUVmean, volumetric parameters of the primary lesion, such as Receptor-Expressing Tumor Volume (RETV) and Total Lesion Receptor Expression (TLRE), volumetric parameters of the lesions in the whole-body, such as wbRETV and wbTLRE, and texture features such as Coefficient of Variation (CoV), HISTO Skewness, HISTO Kurtosis, HISTO Entropy-log_10_, GLCM Entropy-log_10_, GLCM Dissimilarity, and NGLDM Coarseness. Patients were subjected to a mean follow-up period of 17 months, and survival analysis was performed using the Kaplan–Meier method and log-rank tests. **Results**: Forty-seven primary lesions were analyzed. Survival analysis was performed, including clinical variables along with conventional, volumetric, and texture imaging features. At univariate analysis, overall survival (OS) was predicted by age (*p* = 0.0079), grading (*p* = 0.0130), SUVmax (*p* = 0.0017), SUVmean (*p* = 0.0011), CoV (*p* = 0.0037), HISTO Entropy-log_10_ (*p* = 0.0039), GLCM Entropy-log_10_ (*p* = 0.0044), and GLCM Dissimilarity (*p* = 0.0063). At multivariate analysis, only GLCM Entropy-log_10_ was retained in the model (χ^2^ = 7.7120, *p* = 0.0055). Kaplan–Meier curves showed that patients with GLCM Entropy-log_10_ >1.28 had a significantly better OS than patients with GLCM Entropy-log_10_ ≤1.28 (χ^2^ = 10.6063, *p* = 0.0011). **Conclusions**: Texture analysis of 68Ga-DOTATOC PET/CT images, by revealing the heterogeneity of somatostatin receptor expression, can predict the clinical outcome of NET patients.

## 1. Introduction

Neuroendocrine neoplasms are an uncommon, complex, and heterogeneous group of tumors originating from neuroendocrine cells, primarily affecting the gastroenteropancreatic district, but also the bronchopulmonary tract and other organs [1,2]. These tumors can be classified based on their cell morphology and proliferation index (Ki-67) into well-differentiated neuroendocrine tumors (NETs) and poorly differentiated neuroendocrine carcinomas. Therefore, their clinical behavior is quite variable, ranging from indolent and slow-growing to highly aggressive with poor prognosis [3,4]. Moreover, neuroendocrine tumors can also be categorized as functional, with symptoms due to hormone secretion, or non-functional, with asymptomatic presentation or non-specific symptoms that can lead to diagnostic delay [5]. The combination of all these variables in terms of different morphology, biological behavior, and clinical presentation makes the management of neuroendocrine tumors quite difficult, thus underlining the need for effective tools to examine all the different aspects of these tumors. Therefore, the management of NET patients requires a multidisciplinary approach that includes clinical evaluation, biochemical and histopathological examinations, and multimodal imaging methodologies.

Imaging is fundamental in the complex management of NET patients, and among all the available methodologies, receptor imaging with radiolabeled somatostatin analogues has a primary role [6]. The use of this imaging methodology is based on the peculiar characteristic of NETs to overexpress somatostatin receptors (SSTRs), especially if well-differentiated. These receptors are G-protein-coupled transmembrane proteins that, on binding with their specific ligands, modulate cellular proliferative and secretory activity. Their expression can be influenced by several factors, such as the organ affected, tumor differentiation, microenvironmental conditions, genetic mutations, and epigenetic modifications [7,8]. Among the five known somatostatin receptor subtypes, SSTR2 is the most frequently overexpressed in NETs, followed by SSTR5 and SSTR3 [9,10].

Receptor imaging with radiolabeled somatostatin analogues performed by PET/CT with gallium-labeled somatostatin analogues (68Ga-peptide PET/CT) has a pivotal role in the management of NET patients, being of use in all phases of the disease [11]. In fact, 68Ga-peptide PET/CT has a higher diagnostic accuracy than conventional imaging methods [12]. Moreover, this imaging methodology is crucial for selecting patients as candidates for therapy with labeled or unlabeled somatostatin analogues, for the prediction of response to therapy, and for prognostic assessment [11,13,14]. Furthermore, in recent years, the labeling of somatostatin analogues with β-emitting radioisotopes has allowed the development of a theranostic approach in patients with NETs [15,16].

Over the years, various gallium-PET-based biomarkers have been developed to quantify the amount of SSTRs or other characteristics, such as the heterogeneity of their expression for diagnostic, predictive, and prognostic purposes [17,18]. These biomarkers include conventional parameters commonly used in clinical practice, such as Maximum Standardized Uptake Value (SUVmax), which provides a measure of the focal point with the highest SSTRs expression in a NET lesion. However, SUVmax may not represent the receptor status of the entire lesion. To overcome this limitation, volumetric parameters such as Gallium-PET-based Receptor-Expressing Tumor Volume (RETV) and Total Lesion Receptor Expression (TLRE), which is the product of the lesion RETV and the corresponding Mean Standardized Uptake Value (SUVmean), were developed. These parameters provide a global measure of SSTRs expression within single lesions and, by summing the values of each lesion, in the entire body. Previous studies evaluated RETV and TLRE in therapy monitoring and prognosis of NET patients [19,20,21].

In the last years, the radiomic approach and texture analysis have led to the development of newer features that have the potential of revealing subvisual characteristics such as tumor heterogeneity [22,23]. In fact, SSTRs are not uniformly distributed on NET cells, and their expression can vary between different regions of the same lesion or between different lesions in the same patient. Some studies used texture analysis to evaluate this uneven SSTRs distribution, and how it may affect tumor response to therapy with labeled or unlabeled somatostatin analogues and patient outcome [17,24,25].

In the present study, a radiomic approach was adopted to test whether selected variables derived from texture analysis of 68Ga-DOTATOC PET/CT images on primary tumors, reflecting the heterogeneity of SSTR2 expression, may predict survival in patients affected by neuroendocrine tumors.

## 2. Materials and Methods

### 2.1. Patients

A total of 47 patients (31 men, 16 women; mean age ± SD: 62 ± 14 years; range 29–84 years) with pathologically diagnosed NET were included in the study. All patients were subjected to 68Ga-DOTATOC PET/CT scan at our institution. The study was approved by the institutional Ethics Committee, and an informed consent form was signed by all subjects. The anatomical location of the primary tumor was the gastroenteropancreatic district in 35 patients, the bronchopulmonary district in 8, and other anatomical districts in 4 patients. Tumor grading and Ki67 proliferation index were available in 40 patients. These patients were classified as G1 (n = 13), G2 (n = 21), and G3 (n = 6), while Ki67 was <3% in 13 patients, between 3 and 20% in 21 patients, and >20% in 6 patients. Among the 47 patients studied, 19 patients had primary tumor only, 11 patients had also lymph node involvement, and 17 patients showed distant metastases with or without lymph node involvement. Patient characteristics are shown in Table 1. Thirty-two patients received no previous therapy before the 68Ga-DOTATOC PET/CT scan. Among the remaining patients, previous treatments such as chemotherapy, temozolomide, or everolimus were discontinued at least 6 months before the PET/CT scan. In patients under treatment with somatostatin analogues using the standard regimen (30 mg i.m. once every 4 weeks), therapy was discontinued one month prior to the Gallium scan, except when discontinuation of therapy was not clinically recommended. OS was calculated as the time between the PET/CT examination and the date of death.

### 2.2. 68Ga-DOTATOC Labeling

The SomaKit TOC (Novartis Farma s.p.a., Milan, Italy) was used to prepare the radiopharmaceutical. The kit contained the somatostatin analogue DOTATOC (edotreotide), which has a high affinity for SSTR2. The manufacturer’s instructions were carefully followed for edotreotide labeling. In summary, 68Ga-chloride was eluted from a 68Ge/68Ga generator (Eckert & Ziegler Radiopharma GmbH, Berlin, Germany). The radionuclide obtained was then added to 40 μg of edotreotide. The solution obtained was buffered and heated (95 °C, 7 min) and then cooled to room temperature before use. All the procedures were performed under sterile conditions. Thin-layer chromatography was performed to verify labeling efficiency. In all labelings, ≤2% of free 68Ga and ≤3% of colloidal 68Ga were obtained.

### 2.3. 68Ga-DOTATOC Study

Patients underwent a PET/CT scan 60 min after intravenous administration of 68Ga-DOTATOC (135 ± 25 MBq) using an Ingenuity TF scanner (Philips Healthcare, Best, The Netherlands). The CT scan was acquired using the following parameters: 120 kV, 80 mA, 0.8 s rotation time, pitch of 1.5. The PET scan was acquired in 3D mode, from the top of the skull to the upper thigh (3 min/each bed position) from 6 to 8 bed positions per patient, depending on height. An ordered subsets/expectation maximization algorithm was used for iterative reconstruction of images. Filtered back projection of CT reconstructed images was used to obtain attenuation-corrected emission data. Ingenuity TF software (IntelliSpace Portal V5.0, Philips Healthcare, Best, The Netherlands) was used to preliminarily examine the resulting transaxial, sagittal, and coronal PET, CT, and fusion images.

### 2.4. 68Ga-DOTATOC Image Analysis

**Figure 1:**
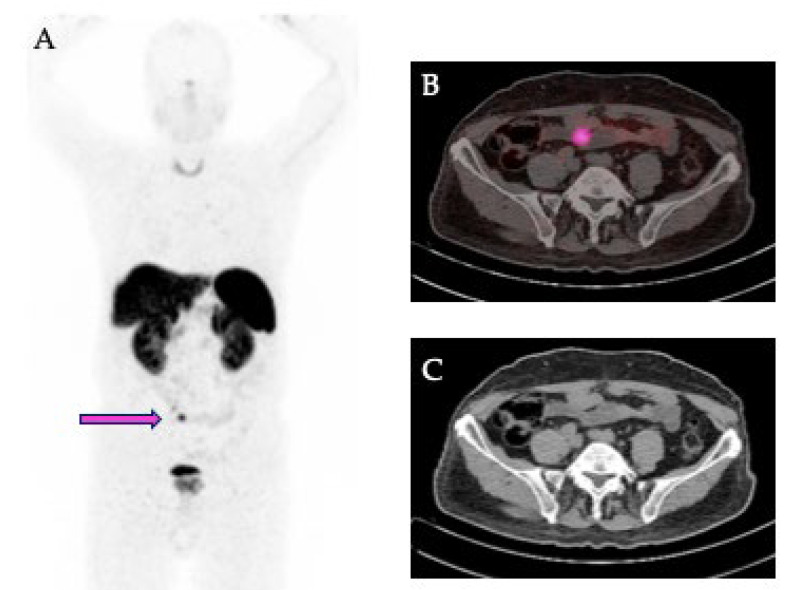
Representative images of a 68Ga-DOTATOC PET/CT scan in a patient with NET of the ileum. (**A**) Maximal intensity projection view showing the primary tumor in the ileum (pink arrow). (**B**) Transaxial fusion image showing the segmentation (pink) of the primary tumor. (**C**) Corresponding transaxial CT image.

The PET/CT data in DICOM format were analyzed by LIFEx software (developed at CEA, Orsay, France, http://www.lifexsoft.org, last accessed on 20 May 2025) [26], obtaining a volume of interest (VOI) of each primary lesion. For this purpose, a three-dimensional region was drawn around each lesion by using an automatic segmentation method that groups all spatially connected voxels within a predetermined threshold. A threshold of SUV > 2.5 was used, based on the mean SUVmax of the mediastinal blood pool plus 2 SD (Figure 1).

In addition, the accuracy of tumor delineation was confirmed on the corresponding CT images. PET variables, including conventional, volumetric, and textural features, were extracted using the LIFEx package (developed at CEA, Orsay, France, http://www.lifexsoft.org, last accessed on 20 May 2025). VOIs that did not reach the minimum number of 64 voxels were excluded from the analysis to avoid inaccurate quantification of texture features inside small lesions. Tonal discretization of gray scale for PET images was adjusted using 64 gray levels with an absolute scale bound between 0 and 100 SUV. Therefore, by computed analysis of each VOI, 46 features (39 texture features and 7 conventional parameters) were extracted. In particular, we included in the analysis conventional parameters such as SUVmax and SUVmean and volumetric parameters such as RETV and TLRE of the primary lesion and whole-body RETV (wbRETV) and whole-body TLRE (wbTLRE) calculated by summing the RETV or TLRE of all lesions present in each patient. Among texture features, we selected variables that, in previous studies [27,28,29], had shown sufficient robustness and repeatability. We selected 4 first-order texture features (Coefficient of Variation (CoV), Histogram Skewness (HISTO Skewness), Histogram Kurtosis (HISTO Kurtosis), and Histogram Entropy-log_10_ (HISTO Entropy-log_10_)) and 3 higher-order texture features (Gray Level Co-Occurrence Matrix Entropy-log_10_ (GLCM Entropy-log_10_), Gray Level Co-Occurrence Matrix Dissimilarity (GLCM Dissimilarity), and Neighborhood Gray-Level Difference Matrix Coarseness (NGLDM Coarseness)) and included them in the analysis.

### 2.5. Statistical Analysis

MedCalc software for Windows, version 10.3.2.0 (MedCalc Software, Mariakerke, Belgium), was used to perform statistical analysis. A probability value < 0.05 was considered statistically significant. Pearson’s correlation coefficient was used to evaluate the linear relationship between continuous variables. Student’s t-test was used to compare means of unpaired data. Univariate and multivariate analyses of clinical and imaging variables were performed using Cox proportional hazards regression. Variables that were able to predict OS by univariate analysis were included in the multivariate analysis. The best discriminative threshold of independent prognostic variables for OS was obtained by receiver operating characteristic (ROC) curve analysis. Survival analysis was performed using the Kaplan–Meier method and log-rank tests. Survivors were censored at the time of the last clinical control.

## 3. Results

We evaluated by 68Ga-DOTATOC PET/CT image analysis 47 primary NET lesions arising from the gastroenteropancreatic (n = 35), bronchopulmonary (n = 8), and other anatomical districts (n = 4). In particular, conventional and volumetric imaging parameters such as SUVmax, SUVmean, RETV, and TLRE, along with the first-order texture variable CoV, were obtained from 47 primary lesions. The average values of these variables were 30.14 ± 28.65, 9.29 ± 7.30, 0.55 ± 0.24, 39.53 ± 66.32 mL, 425.30 ± 801.07 g, respectively (Table 2).

Three first-order texture variables (HISTO Skewness, HISTO Kurtosis, and HISTO Entropy-log_10_) and three higher-order features (GLCM Entropy-log_10_, GLCM Dissimilarity, and NGLDM Coarseness) were extracted from the analysis of 37 primary tumors. In fact, 10 lesions were excluded from analysis due to their volume; ≤64 voxels were too small to allow the extraction of texture features. The average values of these features were 1.17 ± 0.47, 4.07 ± 1.48, 0.95 ± 0.35, 1.76 ± 0.61, 3.12 ± 2.66, and 0.02 ± 0.01, respectively (Table 2). Moreover, wbRETV and wbTLRE were also determined. These parameters were calculated by summing the RETV and TLRE of each lesion in the whole body of each patient, respectively. Therefore, a total of 161 lesions were analyzed, including 47 primary tumors, 29 metastatic lymph nodes, and 85 distant metastases. The mean ± SD of these latter two variables was 106.04 ± 178.53 mL and 1588.19 ± 3954.70, respectively, as reported in Table 2.

After a mean follow-up period of 17 months (mean ± SD: 17 ± 12; range: 1–40 months), 6 patients died and 41 were still alive. The univariate analysis for OS was performed by including age, gender, grading of primary lesion, PET-derived conventional parameters (SUVmax and SUVmean), volumetric variables (RETV and TLRE), whole-body volumetric parameters (wbRETV and wbTLRE), and first- and higher-order texture features (CoV, HISTO Skewness, HISTO Kurtosis, HISTO Entropy-log_10_, GLCM Entropy-log_10_, GLCM Dissimilarity, and NGLDM Coarseness). The variables that significantly predicted OS were age (χ^2^ = 7.0610, *p* = 0.0079), grading (χ^2^ = 6.1630, *p* = 0.0130), SUVmax (χ^2^ = 9.8830, *p* = 0.0017), SUVmean (χ^2^ = 10.7180, *p* = 0.0011), CoV (χ^2^ = 8.4210, *p* = 0.0037), HISTO Entropy-log_10_ (χ^2^ = 8.3500, *p* = 0.0039), GLCM Entropy-log_10_ (χ^2^ = 8.1250, *p* = 0.0044), and GLCM Dissimilarity (χ^2^ = 7.4750, *p* = 0.0063), as shown in Table 3.

**Figure 2:**
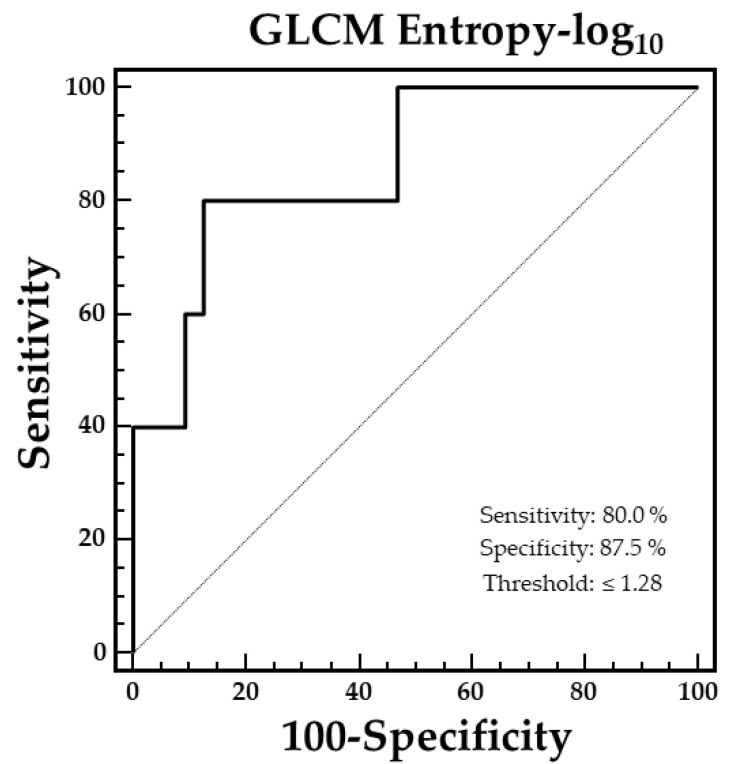
ROC curve analysis showing the optimal GLCM Entropy-log10 value for discriminating between patients who had died and survivors. The threshold determined was 1.28 (AUC = 0.86).

At multivariate analysis, only GLCM Entropy-log_10_ was retained in the model for the prediction of OS (χ^2^ = 7.7120, *p* = 0.0055). A threshold for GLCM Entropy-log_10_ was estimated by ROC curve analysis to discriminate patients who had died from survivors, and a cut-off value of 1.28 (AUC = 0.86) was found (Figure 2).

**Figure 3:**
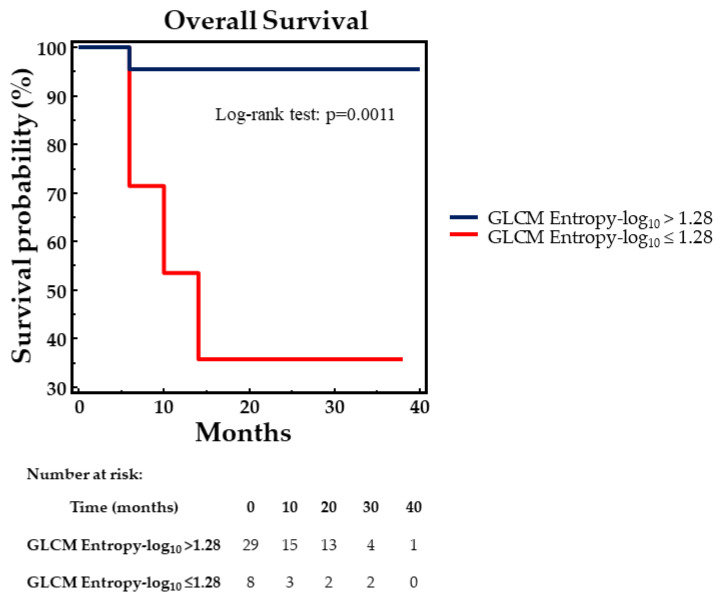
Overall survival by Kaplan–Meier analysis and log-rank test at 17-month follow-up. Statistically significant difference in OS between NET patients with GLCM Entropy-log10 values lower or higher than the cut-off level of 1.28, as assessed by ROC curve analysis (*p* = 0.0011).

Using Kaplan–Meyer analysis and log-rank testing, OS was significantly better in patients with GLCM Entropy-log_10_ > 1.28 as compared to those with GLCM Entropy-log_10_ ≤ 1.28 (χ^2^ = 10.6063, *p* = 0.0011) (Figure 3).

Moreover, we evaluated the correlation between GLCM Entropy-log_10_ and other significant variables and found that GLCM Entropy-log_10_ was significantly correlated with SUVmax (*r* = 0.7382, *p* > 0.0001), SUVmean (*r* = 0.7005, *p* > 0.0001), and CoV (*r* = 0.7434, *p* > 0.0001), while inversely correlated with grading (*r* = −0.4549, *p* = 0.0089). Finally, we performed the Student’s t-test to compare means of GLCM Entropy-log_10_ between G1, G2, and G3, and between survivors and patients who had died. A statistically significant difference was found between the GLCM Entropy-log_10_ of G1 vs. G3 (2.09 ± 0.39 vs. 1.2 ± 0.67, *p* = 0.0049) as well as between survivors and patients who had died (1.88 ± 0.55 vs. 1.05 ± 0.53, *p* = 0.0036).

## 4. Discussion

In our study, we evaluated the heterogeneity of SSTR2 expression in NET patients by analyzing 68Ga-DOTATOC PET/CT images using a radiomic approach in order to select texture variables with prognostic significance. Among all clinical and imaging variables tested, univariate analysis showed that age, SUVmax, SUVmean, CoV, HISTO Entropy-log_10_, GLCM Entropy-log_10_, and GLCM Dissimilarity were predictive of OS, while at multivariate analysis, only GLCM Entropy-log_10_ was an independent predictive factor of survival. Indeed, Kaplan–Meier analysis showed that high entropy levels were significantly associated with better OS compared to low entropy values.

In the latest years, radiomic analysis of PET/CT images has been used for various purposes such as radiogenomics studies, identification of occult lesions, evaluation of tumor staging and grading, differential diagnosis of tumor histotypes, and, finally, for prognostic purposes [24,30,31].

In the daily routine, SUVmax is the most widely used parameter to evaluate PET/CT exams. The biological significance of this conventional parameter varies depending on the tracer; when using 68Ga-peptide PET/CT for the evaluation of NET tumors, SUVmax is an index of SSTR2 expression, and its monitoring over time reflects the modulations that receptor expression may undergo due to therapy or tumor progression. PET/CT image analysis also allows obtaining volumetric parameters, which represent the tumor burden in the primary lesion or in the entire body, by summing the volumes of all focal lesions [32]. However, these parameters do not provide information about the spatial distribution of the tracer within a selected volume. Such spatial information that derives from the intensity ratios of the voxels included in the selected volume may reflect peculiar biological characteristics of the tumor, which may influence the response to therapy or prognosis.

Texture analysis aims to determine the spatial variations in tracer distribution within the tumor volume and to quantify the heterogeneity of tracer uptake within this volume [33,34]. The clinical significance of texture variables and how they may reflect specific tumor biological characteristics or how they may influence the response to therapy or the clinical outcome are still debated issues. Among the possible applications in the management of neoplastic patients, and NET patients in particular, texture analysis may also potentially complement conventional clinical assessment and staging systems in discriminating between well and poorly differentiated tumors, in supporting diagnosis or therapy decisions, or in risk-stratification of patients within the same stage of disease.

Previous PET/CT studies evaluated the prognostic value of texture features in patients with different tumor types [34,35,36,37,38]. Some of these studies performed texture analysis of 68Ga-peptide PET/CT images in NET patients, showing that several texture variables, including Correlation and SZE (Short Zone Emphasis) [39], Skewness and Kurtosis [40], and entropy and homogeneity [39,41,42,43], were able to predict survival. In particular, Werner et al. [39,43] analyzed 68Ga-peptide PET/CT images of NET patients prior to PRRT, finding that entropy, along with other variables, was significantly correlated with survival. This study, in agreement with our results, showed that higher entropy values were associated with a better prognosis.

Furthermore, we found an inverse correlation between GLCM Entropy-log_10_ and grading; indeed, lower GLCM Entropy-log_10_ values, reflecting poor prognosis, were significantly correlated with higher grading, an index of tumor aggressiveness, providing a hint on the possible biological meaning of this second-order texture variable.

Finally, it is worth noting that the relatively small number of patients prevents validation of our findings on an independent dataset that would increase the statistical power of the analysis. Therefore, our results may need confirmation in a multicenter study including external validation. Moreover, the limited sample size does not allow the application of more sophisticated methods such as machine learning and deep learning analyses. However, since neuroendocrine tumors are rare and heterogeneous, a critical issue may be the recruitment of a sufficiently large and homogeneous number of patients to perform this type of analysis. Despite the limited population and the use of a simple statistical approach, our study may provide useful information for developing more sophisticated models that may have a higher clinical impact in the future.

## 5. Conclusions

Our study showed that a second-order texture variable, such as GLCM Entropy-log_10_, is an independent predictive factor of OS in NET patients, by allowing the evaluation of the heterogeneity of somatostatin receptors expression, which reflects the tumor biological characteristics. Therefore, entropy or other texture variables derived by PET/CT image analysis may provide valuable additional information in assessing tumor stage, in evaluating the response to therapy with both cold and radiolabeled somatostatin analogues, and in predicting the clinical outcome. Further studies are needed to consolidate the robustness, standardization, and reproducibility of texture variables to allow their future use in the management of cancer patients, contributing to the development of personalized medicine.

## Figures and Tables

**Table 1:** Clinical characteristics, grading, and staging of 47 patients with neuroendocrine tumors.

Characteristic	N° (%)
Patients	47
**Age**	
Mean ± SD	62 ± 14 years
Range	29–84 years
**Gender**	
Female	16 (34%)
Male	31 (66%)
**Type of NET**	
Gastroenteropancreatic	35 (74%)
Broncopulmonary	8 (17%)
Other	4 (9%)
**Grading**	
G1	13 (27%)
G2	21 (45%)
G3	6 (13%)
Not determined	7 (15%)
**Ki67 (%)**	
<3	13 (27%)
3–20	21 (45%)
>20	6 (13%)
Not Determined	7 (15%)
**Staging**	**Patients**
Primary tumor only	19 (41%)
Primary tumor and metastatic lymph nodes	11 (23%)
Primary tumor and distant metastatic lesions (with or without lymph node involvement)	17 (36%)

**Table 2:** Conventional, volumetric, and texture PET-based imaging features obtained by 68Ga-DOTATOC PET/CT analysis of 47 primary tumors.

Parameters	Mean ± SD	Range
SUVmax	30.14 ± 20.65	4.78–157.23
SUVmean	9.29 ± 7.30	3.29–42.72
RETV (ml)	39.53 ± 66.32	1.5–387.84
TLRE (g)	425.30 ± 801.07	7.3–4776.19
wbRETV (ml)	106.04 ± 178.53	1.5–893.44
wbTLRE (g)	1588.19 ± 3954.70	7.3–21,534.20
CoV	0.55 ± 0.24	0.17–1.00
HISTO Skewness	1.17 ± 0.47	0.44–2.15
HISTO Kurtosis	4.07 ± 1.48	2.15–7.97
HISTO Entropy-log_10_	0.95 ± 0.35	0.31–1.60
GLCM Entropy-log_10_	1.76 ± 0.61	0.55–2.95
GLCM Dissimilarity	3.12 ± 2.66	0.36–10.91
NGLDM Coarseness	0.02 ± 0.01	0.001–0.069

Standard Deviation (SD); Receptor-Expressing Tumor Volume (RETV); Total Lesion Receptor Expression (TLRE); whole-body RETV (wbRETV), whole-body (wbTLRE); Coefficient of Variation (CoV).

**Table 3:** Predictors of overall survival by univariate analysis of clinical and imaging variables.

Variables	Overall Survival	
	χ^2^	*p*
Age	7.0610	0.0079
Gender	0.0329	0.8562
Grading	6.1230	0.0130
SUVmax	9.8830	0.0017
SUVmean	10.7180	0.0011
RETV	0.8030	0.3703
TLRE	3.2820	0.0700
wbRETV	0.0000123	0.9972
wbTLRE	0.7200	0.3962
CoV	8.4210	0.0037
HISTO Skewness	2.2160	0.1366
HISTO Kurtosis	1.5430	0.2142
HISTO Entropy-log_10_	8.3500	0.0039
GLCM Entropy-log_10_	8.1250	0.0044
GLCM Dissimilarity	7.4750	0.0063
NGLDM Coarseness	0.2210	0.6381

Receptor-Expressing Tumor Volume (RETV); Total Lesion Receptor Expression (TLRE); whole-body RETV (wbRETV), whole-body (wbTLRE); Coefficient of Variation (CoV).

## Data Availability

The original contributions presented in this study are included in the article. Further inquiries can be directed to the corresponding author.

## Abbreviations

The following abbreviations are used in this manuscript:

68Ga-DOTATOC PET/CT	68Gallium-DOTATOC Positron Emission Tomography/Computed Tomography
NET	Neuroendocrine Tumors
SUVmax	Maximum Standardized Uptake Value
SUVmean	Mean Standardized Uptake Value
RETV	Receptor-expressing Tumor Volume
TLRE	Total Lesion Receptor Expression
wbRETV	Whole-body Receptor-expressing Tumor Volume
wbTLRE	Whole-body Total Lesion Receptor Expression
CoV	Coefficient of Variation
HISTO Skewness	Histogram Skewness
HISTO Kurtosis	Histogram Kurtosis
HISTO Entropy-log_10_	Histogram Entropy-log_10_
GLCM Entropy-log_10_	Gray Level Co-Occurrence Matrix Entropy-log_10_
GLCM Dissimilarity	Gray Level Co-Occurrence Matrix Dissimilarity
NGLDM Coarseness	Neighborhood Gray-Level Difference Matrix Coarseness
OS	Overall Survival
SSTRs	Somatostatin Receptors
VOI	Volume of Interest
SD	Standard Deviation
ROC	Receiver-Operating Characteristic
SZE	Short Zone Emphasis
